# Cardiometabolic adverse effects of long-term antipsychotic treatment in children and adolescents with non-psychotic disorders: a systematic review of available evidence

**DOI:** 10.1007/s00787-025-02771-0

**Published:** 2025-06-05

**Authors:** Ramya Padmavathy Radha Krishnan, Monika Dzidowska, Danni Zheng, Zoie Shui-Yee Wong, Nicholas A. Buckley, Jacques Eugene Raubenheimer

**Affiliations:** 1 Faculty of Medicine and Health, The University of Sydney, RC Mills Building Room 107, Sydney, NSW 2006 Australia; 2 Graduate School of Public Health, St. Luke’s International University, Tokyo, Japan

**Keywords:** Antipsychotic, Off-label disorders, Cardiovascular and metabolic adverse effects, Safety outcomes, Non-psychotic conditions, Children and adolescents

## Abstract

Antipsychotics are known to cause cardiovascular and metabolic adverse effects, but there is less evidence for their use off-label, especially in children and adolescents. Such treatments may be administered over long durations. We conducted a systematic review to examine the cardiometabolic adverse effects of prolonged antipsychotic treatment in children and adolescents with non-psychotic disorders. We searched for randomised clinical trials and observational studies lasting for a year or more, examining the adverse outcomes of weight gain, hyperglycaemia, dyslipidaemia, hypertension, metabolic syndrome, ischaemic heart disease, and thrombosis. There were no randomised trials, and 15 observational studies involved 114,141 participants with mean age of 10.9 years (range of means 4.3–14.9 years). Twelve antipsychotics were covered but the majority (12 studies, 80%) examined risperidone. Using the vote counting method, long-term antipsychotic treatment was associated with weight gain (91.6% studies, *n* = 12), hyperglycaemia (100%, *n* = 6), dyslipidaemia (66.6%, *n* = 6), and metabolic syndrome (100%, *n* = 2). Hypertension was demonstrated in only one study (25%, *n* = 4), and none examined ischaemic heart disease or thrombosis. Studies exhibited moderate to high methodological quality. Prolonged exposure to antipsychotics during childhood may be associated with several metabolic adverse effects that could have long-term health impacts. Longer-term studies on a wider range of agents are required to clarify risks and develop appropriate strategies to mitigate them. Antipsychotic prescribing for children should be assessed on an individual basis, after weighing the benefits of therapy against the harms that may arise. Children need to be monitored for these metabolic effects, and early intervention is essential if adverse effects are severe.

**Supplementary Information:**

The online version contains supplementary material available at 10.1007/s00787-025-02771-0.

## Introduction

Antipsychotic use has increased globally across all age groups, including use in both licensed and unlicensed indications [1–4]. Among children and adolescents, antipsychotic agents and their licensed indications differ across countries but commonly include schizophrenia, bipolar I disorder, treatment of persistent aggression in conduct disorder (risperidone), irritability associated with autism (haloperidol, risperidone, aripiprazole), and Tourette’s syndrome (aripiprazole, pimozide, haloperidol) [5]. Off-label use in this age group may consist of use of agents in unlicensed indications, use at greater doses or duration, and use other than the specified age, leading to efficacy and tolerability issues [6]. Off-label prescribing rates vary from 36–93.2% in children and adolescents, with an increase over the years in both incidence and prolonged use [3, 7, 8]. Predominant indications for off-label antipsychotic therapy in this age group are conduct disorders (long-term treatment), autism spectrum disorder, attention deficit hyperactivity disorder (ADHD), pervasive developmental disorders, anxiety disorders, oppositional disorders and depression, where they may be used for symptoms such as aggression, anxiety, impulsiveness, irritability, agitation, disruptive behaviours, sleep issues, tics and self-injury [7–10]. Frequently used antipsychotics in children are risperidone, quetiapine, olanzapine and aripiprazole among the second-generation (SGAs) and haloperidol, pimozide and pipamperone among the first-generation antipsychotics (FGAs) [7, 9, 10].

SGAs have gained in popularity since their development due to exhibiting fewer neurological adverse events such as extrapyramidal symptoms but have the propensity to cause cardiovascular (hypertension, coronary heart disease, sudden cardiac death), metabolic (diabetes, weight gain, dyslipidaemia), and cerebrovascular disturbances in adults with psychotic illnesses [11]. Considerable variability exists between agents and FGAs can also cause these metabolic changes, thus challenging this distinction between FGAs and SGAs [8, 12]. Children and adolescents seem to be more susceptible to metabolic disturbances including weight gain than adults. Other adverse effects in adolescents include hyperprolactinaemia, sexual dysfunction, arrhythmias and increased risk of unexpected death [8, 12, 13]. There are limited studies examining these effects in off-label indications, and the few systematic reviews that have explored these issues have not differentiated between long-term and short-term effects [14–16]. However, in paediatrics, the majority of use is off-label, long-term and involves psychotropic comedication [3, 17, 18]. With the possibility of symptom recurrence with antipsychotic tapering or discontinuation [8], there is a need for clear evidence on long-term adverse effects, especially when started in childhood or adolescence, to understand the risks/benefits of this increasing off-label use. These concerns were raised in a recent umbrella review that failed to find adequate data on the impact of long-term treatment duration on metabolic adverse effects [19].

Herein we conducted a systematic review to synthesise the evidence from randomized clinical trials and observational studies of cardiometabolic adverse effects arising from long-term antipsychotic treatment in children and adolescents with non-psychotic indications. This study is part of a wider systematic review encompassing all age groups.

## Methods

### Study design

This systematic review was conducted according to the Preferred Reporting Items for Systematic Reviews and Meta-Analyses (PRISMA) guidelines [20]. We registered the review protocol for the wider systematic review with Prospero (CRD42022295571) on 1 st January 2022 prior to full-text screening [21]. The PICOT (Population, Intervention, Comparison, Outcome, Timeframe) strategy was employed to develop the eligibility criteria.

English-language peer-reviewed publications of randomised clinical trials and observational studies in humans (cohort, cross-sectional and case–control) with any comparator were included, with no limitations on publication date. Non-English publications were excluded due to the unavailability of translation services. All non-psychotic illnesses were considered. Studies that included arms with both psychotic and non-psychotic indications (mixed population groups) were excluded due to confounding by the psychotic indication. Treatment with any antipsychotic was allowed, including combination treatments. Treatment duration had to be at least twelve months. The complete list of disorders, antipsychotic agents, outcomes and outcome measures are given in Online Resource 1 (eTable 1).

For the overarching screening, studies on pregnant women were excluded. Though initially included in the protocol, patients with eating disorders were later excluded as it is challenging to separate drug effects from physiological changes due to the underlying conditions. All other publication types were excluded.

### Search strategy

Our search strategy included the three main themes of antipsychotics, off-label disorders, and the adverse events of interest (Online Resource 1, eTable 2). We did not limit searches by treatment duration or study type to avoid missing important studies. The search strategy was applied to the electronic databases – Medline, Embase, PsycINFO, CINAHL, Web of Science, and Scopus. Additional articles were collected through the citation lists of relevant reviews. The initial search was completed in Dec 2021 and searches were periodically rerun to include newly published articles until 10 th July 2024. All references were collected using EndNote software and duplicates were removed.

### Screening

The initial search yielded over 77,000 references due to the large number of disorders, interventions and outcomes (Online Resource 1, eTable 1). After removing duplicates, we used EndNote’s Smart Group and Search functionality to perform title screening and eliminated nearly 97% of the inappropriate hits (details in Online Resource 1, eTable 3). The standard dual-reviewer (JR and RK) approach was employed as a pre-screening process for a random 5% sample of studies to establish consensus and minimise errors. Abstract screening for the remaining references (n = 4,782) was then carried out in Covidence by a single reviewer (RK). Full-text screening of each article was performed by two independent reviewers (RK, JR, MD, or DZ, Cohen’s kappa values for initial agreement ranged between 0.47–0.61). Exclusion criteria were recorded, and conflicts resolved through discussion until the reviewers reached unanimous agreement. Where required, we contacted study corresponding authors for more details on missing or unclear information.

### Data extraction

We conducted a pilot extraction by creating data extraction forms in Microsoft Excel, which captured details on the study design, patient characteristics, intervention and comparator details, and outcomes. A single reviewer (RK) then performed data extraction, which was independently verified by another reviewer (JR or ZW). Where data were unclear, RK contacted the study authors for more details. Both dichotomous and continuous data were captured. Values for outcome measures were converted to international standard units. Data for all outcome measures and time points were captured for each outcome, where available. Studies examining multiple antipsychotics were counted individually for each antipsychotic where possible. The OpenMeta[Analyst] application was utilized to compute odds ratios and mean differences [22].

### Quality assessment

Two independent reviewers (RK, JR, or ZW) evaluated the quality of each included study and disagreements were resolved by discussion. We chose the Cochrane Risk of Bias (RoB) tool to evaluate randomized controlled trials, the Newcastle–Ottawa Scale (NOS) to assess cohort and case–control studies [23], and the Joanna Briggs Institute (JBI) critical appraisal tool for cross-sectional studies [24].

### Data synthesis

Due to methodological and clinical heterogeneity across study designs and comparators, we adopted a narrative synthesis according to the Synthesis Without Meta-analysis (SWiM) guideline [25]. Results were synthesised based on the seven main outcomes (weight gain, hyperglycaemia, dyslipidaemia, hypertension, metabolic syndrome, thrombosis and ischaemic heart disease). We employed a vote-counting method to judge results for the outcomes. We used the direction of effect, *without considering* statistical significance or magnitude of the effect estimate, as suggested by the Cochrane Handbook [26]. We classified the direction as showing increase (deterioration of the adverse outcome), decrease (improvement), or no change/inconclusive. Where multiple outcome measures were assessed, a minimum of 65% similarity (demonstrating a clear majority) in the direction of effect was required, failing which we categorized the direction as inconclusive. We developed an effect direction plot using the Microsoft Excel template provided by Boon et al. [27] to graphically summarize the results for each outcome. Forest plots were built for continuous outcome measures with at least three studies. Due to heterogeneous comparators, these were used for representative purposes and overall estimates were not calculated.

## Results

There were 150,244 references identified across all age groups, with over one-third being duplicates. After screening 727 full texts, we included 30 (Online Resource 2, eFigure 1). Among these, 15 studies corresponding to 16 reports focussed on children and adolescents (subjects aged 18 years or below). Two reports that assessed the same population were combined [28, 29]. Study characteristics are summarized in Table 1. There were no randomized clinical trials. Ten cohort (30% prospective), three cross-sectional, and two case–control studies published between 2008–2020 were included, with nearly half conducted in the USA (46.7%). In all, there were 114,141 participants with mean age 10.9 years (range of means 4.3–14.9 years) and 83.4% male. Ten studies (66.6%) examined autism spectrum disorder or Tourette syndrome (five each), while the remaining included participants with various mixed psychiatric disorders (mainly ADHD, disruptive behaviour disorders, anxiety disorder, depression and autism spectrum disorder, details in Online Resource 1 eTable 4). Three (20%) included patients with prior antipsychotic exposure, eight (53.3%) included patients with psychiatric comorbidities, and 12 (80%) studies involved other psychiatric comedications (mainly psychostimulants, antidepressants and alpha-2 agonists, details in Online Resource 1 eTable 5). Baseline differences in characteristics between the intervention and control arms were observed in 11 (73.3%) studies. These included differences in mean age, sex distribution, severity of the indication, and comedications administered to the subjects. Nine studies adjusted for these imbalances in their analyses.

**Table 1:**
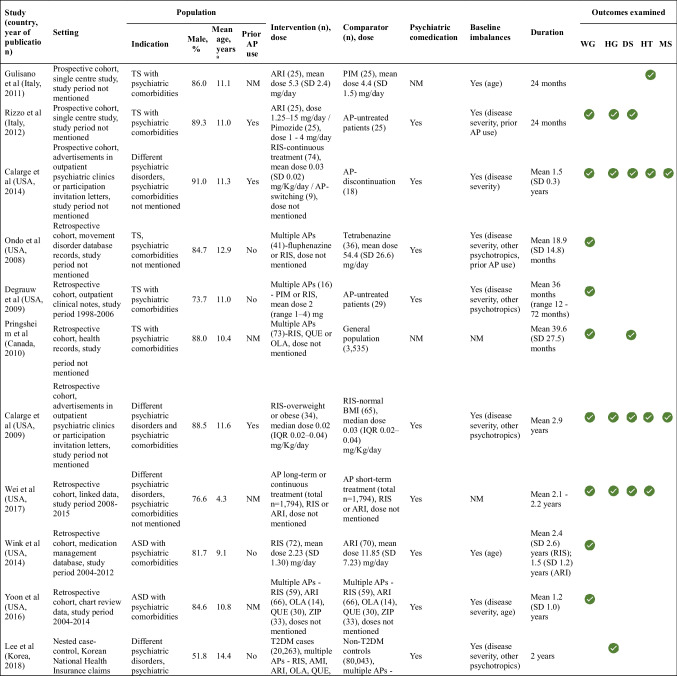

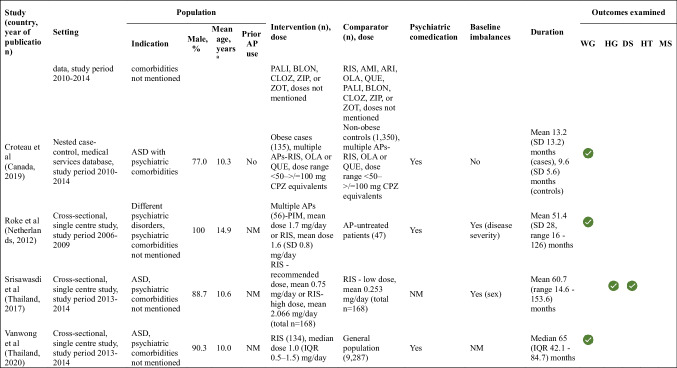
Characteristics of included studies

^a^Mean age was calculated as the average across the cohorts and hence SD is not provided

None of the studies examined ischaemic heart disease or thrombosis, hence these outcomes are not included in the table

*AMI* Amisulpride *AP* antipsychotic *ARI* Aripiprazole *ASD* Autism spectrum disorder *BLON* Blonanserin *BMI* body mass index *CLOZ* Clozapine *CPZ* Chlorpromazine *DS* dyslipidaemia *HG* hyperglycaemia *HT* hypertension *IHD* ischemic heart disease *IQR* inter-quartile range *MS* metabolic syndrome *NM* not mentioned *OLA* Olanzapine *PIM* Pimozide *QUE* Quetiapine *RIS* Risperidone *SD* standard deviation *T2DM* type 2 diabetes mellitus *TH* thrombosis *TS* Tourette syndrome *WG* weight gain *ZIP* Ziprasidone *ZOT* Zotepine

**Fig. 1:**
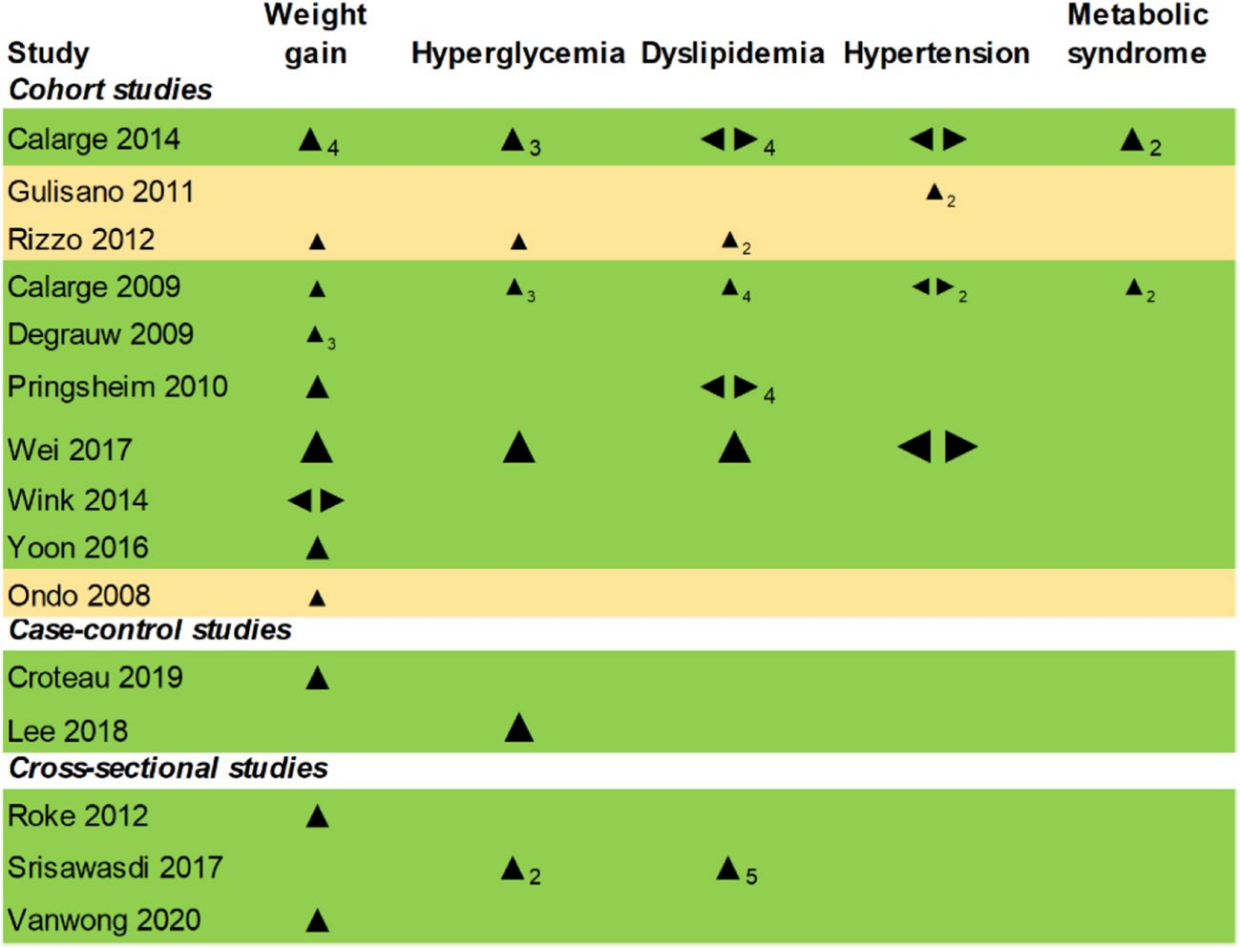
Effect direction plot giving the study-wise direction of effect for each outcome. Study-wise results for each outcome are given. Arrows indicate the direction of effect (upwards for increase, downwards for decrease, and bidirectional for no change/inconclusive). The size of the arrow indicates the final sample size in the intervention group (small for < 50, medium for 50–300, and large for > 300 participants). The subscript denotes the number of measures per outcome if it is more than one. The colour of the row indicates study quality (green for high quality, yellow for some concerns, and red for low quality)

Data was available for 12 antipsychotics (treatment duration range 1–5.5 years), with risperidone being included in 12 (80%). More than half the studies included active comparators (n = 8, 53.4%). Information on outcomes and individual antipsychotic agents is presented in Online Resource 2 (eFigure 2). Results based on the vote-counting method are summarized in Table 2 for each outcome and in the effect direction plot (Fig. 1) for each study. Weight gain had the strongest evidence, followed by hyperglycaemia and to a smaller extent, dyslipidaemia. There was no data for thrombosis and ischaemic heart disease. Individual estimates are provided in Online Resource 3 (eTable 6).

**Table 2:**
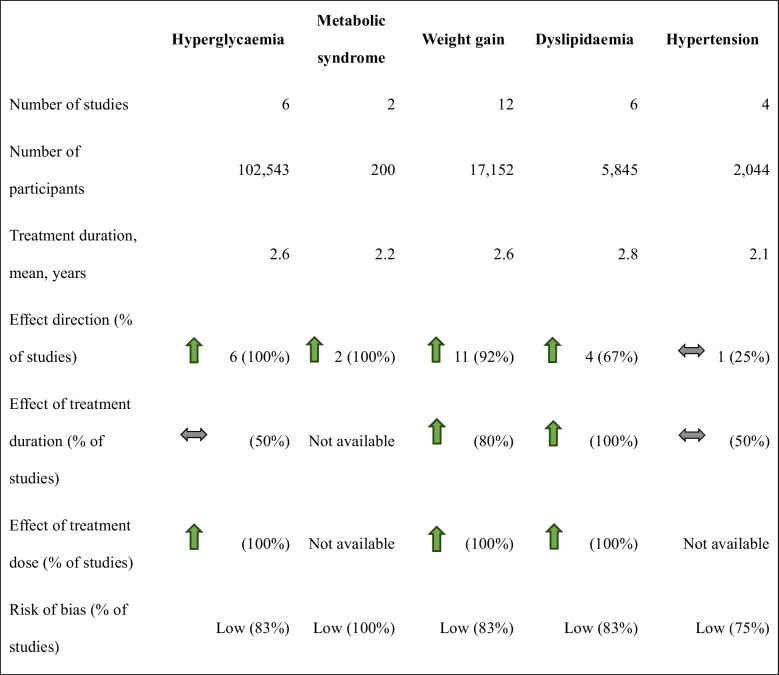
Summary of results for each outcome

Increase (worsening of outcome)

Decrease (better outcome)

Inconclusive or conflicting evidence

### Weight gain

**Fig. 2:**
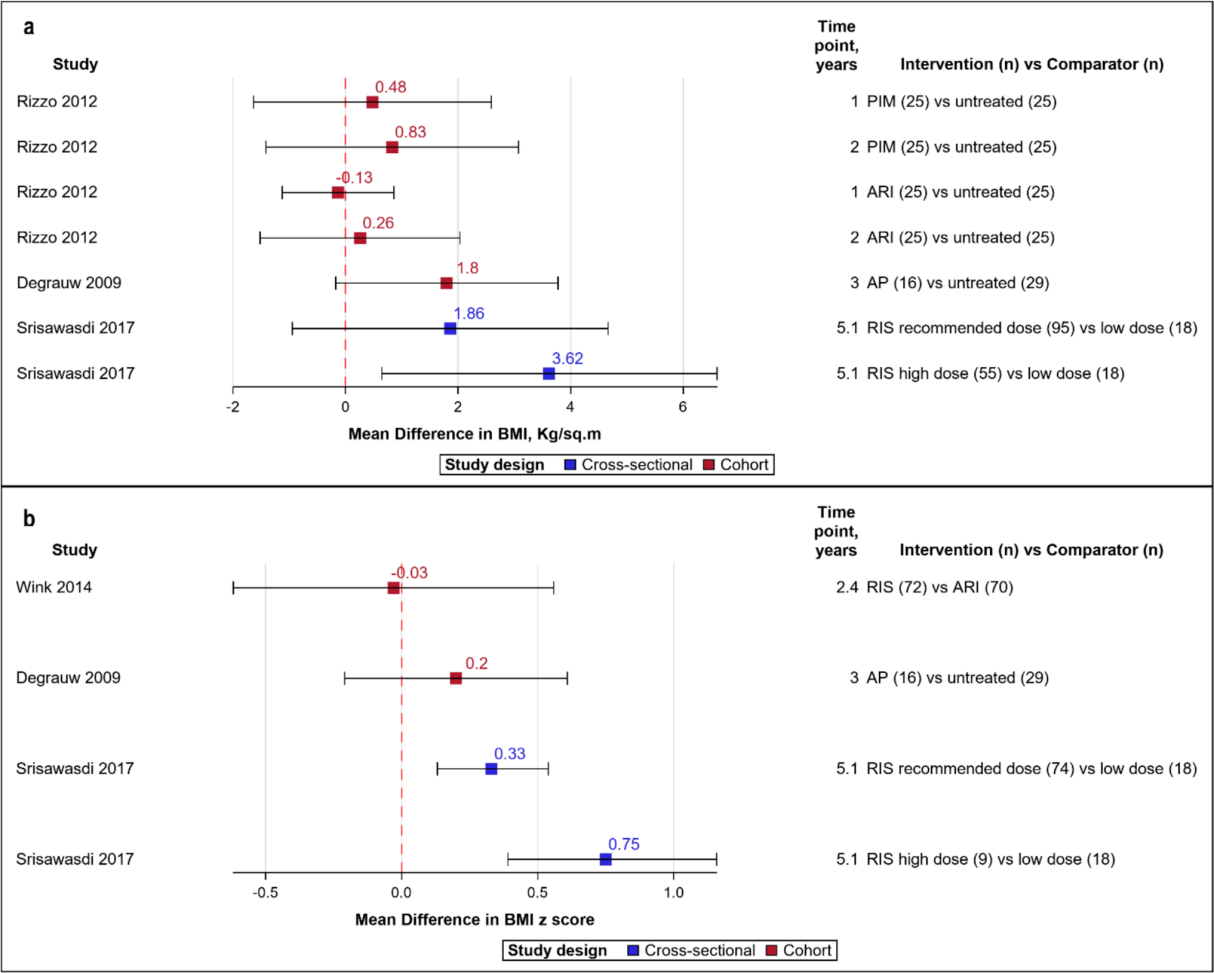
Forest plot for mean differences in (2a) BMI, (2b) BMI z scores. Red dash line indicates 0 or no difference. Red boxes denote cohort studies and blue cross-sectional. Horizontal lines represent the 95% confidence intervals. The study details, measurement time points and the groups being compared are given, with sample size in parentheses. *AP* antipsychotic *ARI* Aripiprazole *BMI* body mass index *HDL* high density lipoprotein *LDL* Low density lipoprotein *PIM* Pimozide *RIS* Risperidone

Twelve (80%) studies that included 17,152 patients examined variations in weight after treatment (Table 2) [28, 30–40]. BMI and BMI z score (derived by comparing to an age and sex-matched reference population) values were the main outcome measures. Among them, 11 (91.7%) studies reported weight gain after treatment, through the vote-counting method (Fig. 1) [28, 30–35, 37–40]. A cohort study reported no difference between risperidone and aripiprazole, with both arms showing increases in BMI and BMI z score [36]. The prevalence of waist circumference >/= 90 th percentile ranged between 10–50% in two studies [28, 34], and the incidence rate of obesity per 1000 person-years was 37.6 with continuous antipsychotic use among preschoolers in one study [35]. The forest plots for mean differences in BMI (Fig. 2a, range −0.13 to 3.62) and BMI z score (Fig. 2b, range −0.03 to 0.75) demonstrated a dose and time-dependent gain in weight. This differed by the agent used, though with wide confidence intervals. One cohort study identified significant BMI z score change compared to baseline after an average of one year of treatment with olanzapine, followed by risperidone and aripiprazole. Quetiapine and ziprasidone showed minimal change [37].

### Hyperglycaemia

**Fig. 3:**
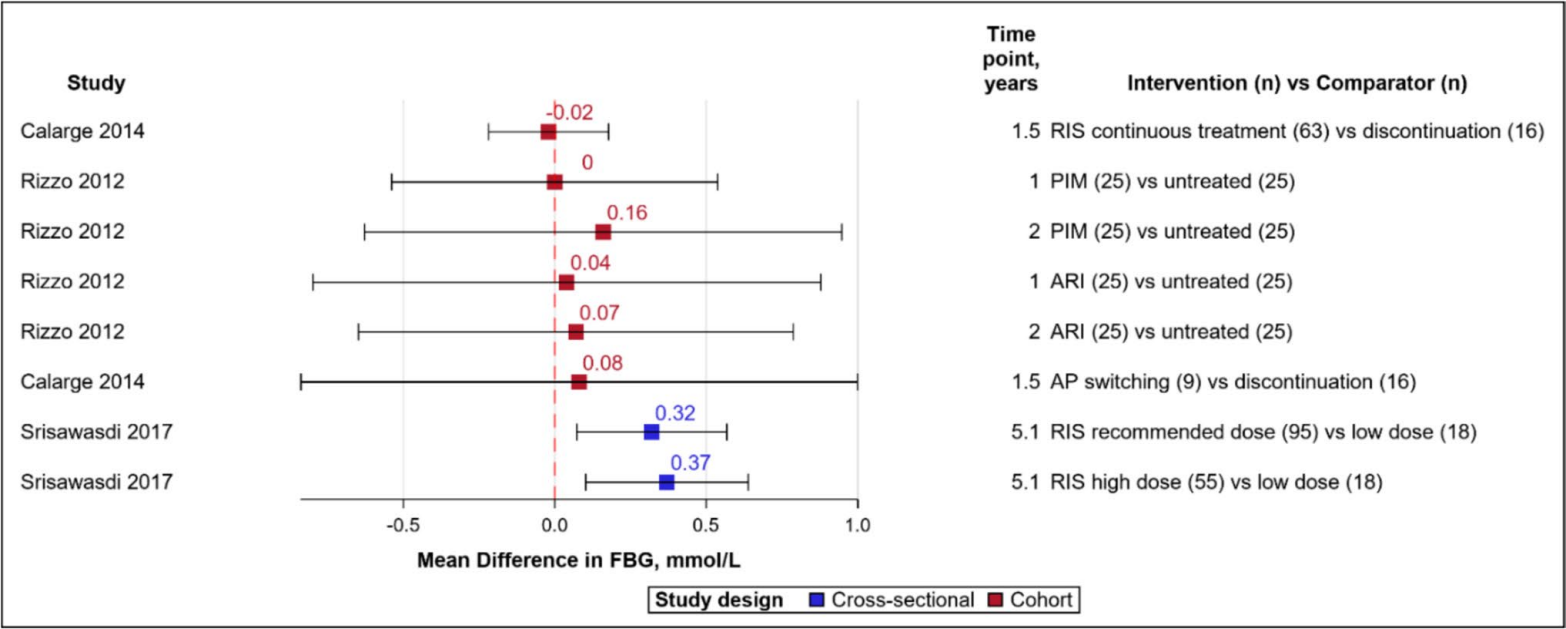
Forest plot for mean differences in fasting blood glucose. Red dash line indicates 0 or no difference. Red boxes denote cohort studies and blue cross-sectional. Horizontal lines represent the 95% confidence intervals. The study details, measurement time points and the groups being compared are given, with sample size in parentheses. *AP* antipsychotic *ARI* Aripiprazole *BMI* body mass index *FBG* fasting blood glucose *HDL* high density lipoprotein *LDL* Low density lipoprotein *PIM* Pimozide *RIS* Risperidone

Six (40%) studies involving 102,543 subjects assessed glucose dysregulation, of which two assessed hyperglycaemia as fasting blood glucose (FBG) >/= 5.6 mmol/L [28, 34], two considered diabetes diagnosis or prescriptions for medicines to lower blood glucose levels [35, 41], and the last two examined increases in FBG and/or insulin levels after treatment [30, 42]. For simplicity, we have collectively used the term “hyperglycaemia” to denote all the above definitions. All six studies (100%) reported increased hyperglycaemia through the vote-counting method (Fig. 1) [28, 30, 34, 35, 41, 42]. Outcome measures included FBG, insulin levels, or Homeostatic Model Assessment for Insulin Resistance (HOMA-IR, an index used to estimate insulin resistance based on FBG and insulin levels, with higher values indicating increased insulin resistance). The prevalence of FBG >/= 5.6 mmol/L and HOMA-IR > 4.39 (threshold definition for high insulin resistance) ranged between 11–22% and 5–25%, respectively, among the two cohort studies that estimated prevalence [28, 34]. The incidence rate of diabetes per 1000 person-years was 4.4 with continuous antipsychotic use among preschoolers in one study [35]. Mean differences in FBG values ranged from −0.02 to 0.37 mmol/L (Fig. 3), many with wide confidence intervals. Treatment dose had a positive association with hyperglycaemia [41, 42]. Among four studies that assessed the effect of treatment duration, two studies (one on preschoolers and the other included subjects with prior antipsychotic use) observed no significant effect [30, 35], the third reported increased risk for type 2 diabetes among adolescents [41] and the fourth study identified increased insulin resistance (without a significant increase in FBG) over a period of 96 months [42]. The third study, which had a nested case–control design, also identified increased adjusted odds ratios for all ten antipsychotics studied, the highest being for clozapine, zotepine and olanzapine (Online Resource 3, eTable 6) [41].

### Dyslipidaemia

**Fig. 4:**
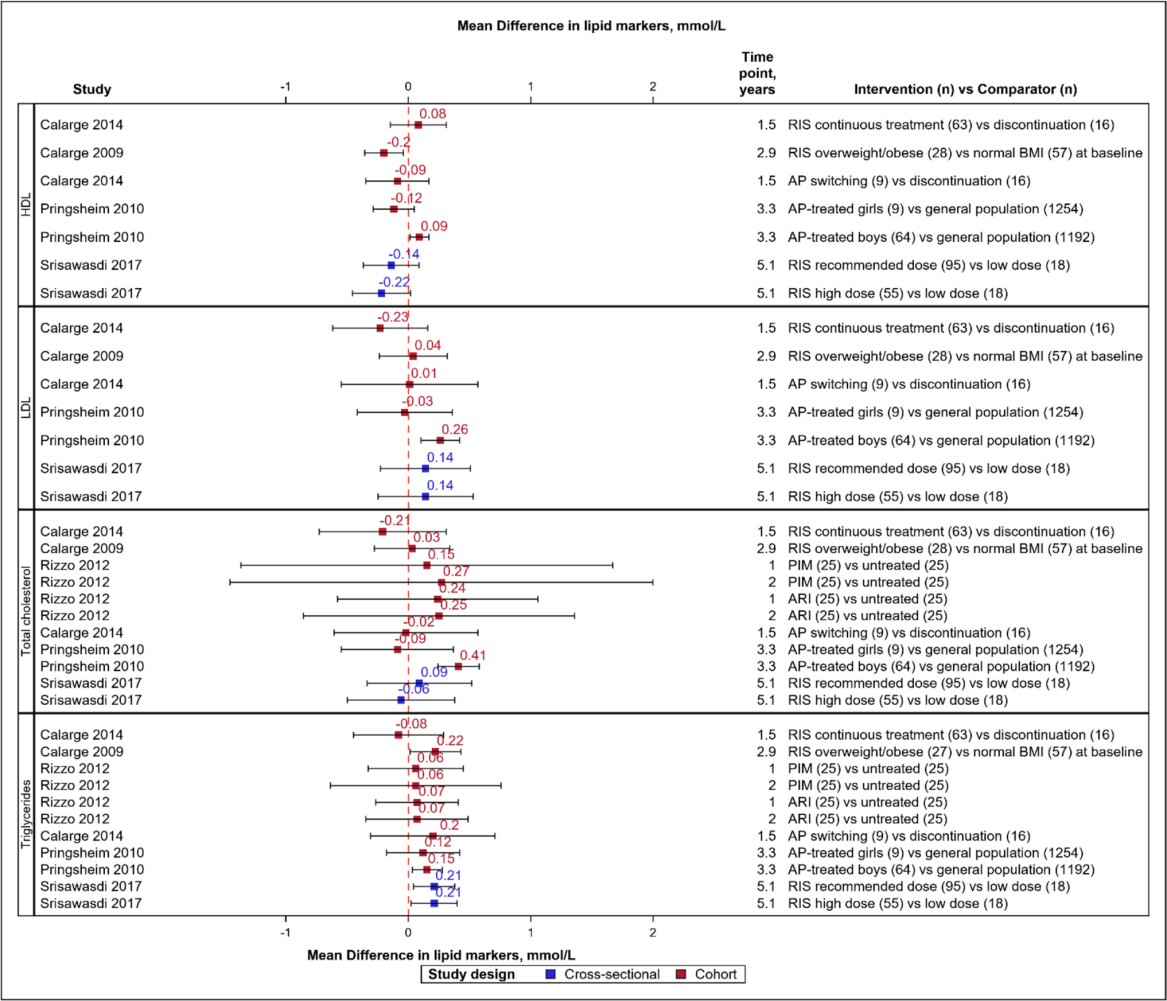
Forest plot for mean differences in blood lipid markers, mmol/L**.** Red dash line indicates 0 or no difference. Red boxes denote cohort studies and blue cross-sectional. Horizontal lines represent the 95% confidence intervals. The study details, measurement time points and the groups being compared are given, with sample size in parentheses. *AP* antipsychotic *ARI* Aripiprazole *BMI* body mass index *HDL* high density lipoprotein *LDL* Low density lipoprotein *PIM* Pimozide *RIS* Risperidone

Six (40%) studies [28, 30, 33–35, 42] with 5,845 subjects investigated dyslipidaemia, and four (66.6%) of these six [30, 34, 35, 42] reported lipid marker alterations (Fig. 1). They observed decreased high-density lipoprotein (HDL) cholesterol levels (−0.09 to −0.22 mmol/L) and increased total cholesterol (0.03 to 0.41 mmol/L) and triglycerides (0.06 to 0.22 mmol/L) levels (Fig. 4). Changes in low-density lipoprotein (LDL) were variable. One cohort study observed variations by sex [33]. The prevalence of triglyceride levels >/= 1.24 mmol/L and HDL cholesterol levels </= 1.034 mmol/L ranged between 14–44% and 17–22%, respectively, in two studies [28, 34]. The incidence rate of dyslipidaemia per 1000 person-years was 7.5 with continuous antipsychotic use among preschoolers in one study [35]. Another study reported duration and dose-dependent alterations in HDL cholesterol and triglycerides [42], while two studies [30, 35] observed increased dyslipidaemia over the initial year, which was sustained at later time points.

### Hypertension

Four (26.7%) cohort studies that together included 2,044 participants examined hypertension [28, 34, 35, 43]. Only one (25%) of these four studies observed an increase in blood pressure with aripiprazole compared to pimozide at both 12 and 24 months of treatment (Table 2 and Fig. 1) [43]. The other three (two involving risperidone) had variable comparators and conflicting findings between treatment arms (Online Resource 3, eTable 6) [28, 34, 35]. The prevalence of systolic or diastolic blood pressure >/= 90 th percentile ranged between 16–44% in two studies [28, 34]. The incidence rate of hypertension per 1000 person-years was 2.9 with continuous antipsychotic use among preschoolers in one study [35].

### Metabolic syndrome

Only two cohort studies assessed the presence of metabolic syndrome in 200 risperidone-treated patients [28, 34], both using the same criteria as defined by Cook et al. [44]. Though having disparate comparators (Online Resource 3, eTable 6), both studies noted increased prevalence of at least one positive metabolic syndrome criterion (as defined in eTable 6) ranging from 21–78%, with a low prevalence of metabolic syndrome (presence of three or more metabolic syndrome criteria, defined in eTable 6) at follow-up (0–22%).

Results of the study quality assessment are presented in Online Resource 2 (eFigure 3). Three cohort studies were of medium (4–6 stars) quality on the NOS scale, losing stars for either not providing patient eligibility information or not adjusting for confounders [30, 31, 43], while the rest were of high quality (7–9 stars). All case–control and cross-sectional studies were deemed to be of high quality using the NOS scale and JBI tool respectively.

## Discussion

This systematic review investigated the cardiometabolic outcomes of prolonged antipsychotic treatment for non-psychotic indications in children and adolescents. A rigorous and comprehensive literature screening was performed to encompass a wide range of non-psychotic disorders, antipsychotic medications and different outcomes. Unlike previous systematic reviews that examined limited parameters [19], we included a range of outcome measures to provide a comprehensive insight into metabolic health in this situation. Fifteen observational studies had sufficient treatment duration to meet our eligibility criteria. Few studies compared treatment outcomes to the general population (*n* = 2, 13.3%) or antipsychotic-untreated patients (*n* = 3, 20%), and five (33.3%) had less than 50 subjects in the intervention group. They all were heterogeneous, which precluded meta-analyses. Outcomes that worsened with treatment were weight gain, hyperglycaemia, dyslipidaemia and metabolic syndrome criteria. Studies were of moderate to high quality.

Most of the efficacy and tolerability data for antipsychotics in children/adolescents are from short-term clinical trials, or observational studies lasting between 6–12 months, whereas treatment is usually required for many years to control symptoms [8, 12, 19]. Weight gain is the most prominent adverse effect in these studies, occurring proximally to antipsychotic initiation. The extent varies by the antipsychotic used, with the maximum weight gain seen to occur with olanzapine [8, 12, 19]. There are similar concerns for the risk of type 2 diabetes among children/adolescents, with a threefold increased incidence compared to a healthy population [45]. The timing for diabetes diagnosis from antipsychotic initiation varied from 4–13.5 months in a few studies, depended on both dose and duration, and varied by the underlying indication and the antipsychotic agent used, similar to weight gain [12]. Along with weight gain and obesity, other mediating factors for diabetes development may include modulation of insulin secretion, insulin resistance, hyperlipidaemia resulting in abnormal glucose metabolism, or impaired pancreatic beta-cell response [12, 14]. Dyslipidaemia is also postulated to occur through both weight-dependent and independent effects [12]. All these metabolic abnormalities can result in the development of metabolic syndrome and cardiovascular morbidity in the long-term [14].

Our review extends these findings to long-term treatments in non-psychotic indications, with dependencies on both dose and duration of therapy. We observed increased prevalence of some of these outcomes (increased waist circumference 10–50%, increased FBG 11–22%, increased triglycerides 14–44%, and decreased HDL 17–22%). In contrast, the global prevalence estimates of obesity and type 2 diabetes in healthy children/adolescents is 8.5% and < 1%, respectively [46, 47]. A surprising exception was the effect of treatment duration on hyperglycaemia (not associated in two of four studies), which can still be attributed to differences between the included studies: a) different age groups studied, with previous studies highlighting the susceptibility of adolescents to diabetes development, b) different indications, c) antipsychotic agent studied, d) prior antipsychotic use (which can introduce survivor bias), and e) psychiatric comedications, with antidepressants increasing the risk [12, 14, 45]. Though there is no universally accepted standard for metabolic syndrome definition in children/adolescents [48], it is concerning to note the high prevalence of at least one metabolic syndrome criterion in the two studies that did examine this outcome, which could have lasting effects well into adulthood. Using the same definition for metabolic syndrome, our study found increased prevalence (up to 22%), as compared to 4% observed among healthy adolescents in a previous study [44]. The absence of studies on ischaemic heart disease and thrombotic events in children is perhaps not surprising given such events are very rare in this age group. A multinational self-controlled case series study (*n* = 48,515) examining both children and young adults (with mean age > 20 years) found increased metabolic effects but not cardiovascular effects [49]. Notably, the type of agents used (FGAs vs SGAs) differed between the countries as did the risks in the former study. It remains to be seen if long-term exposures have a role to play in these two outcomes.

We found no randomized clinical trials in children/adolescents examining metabolic effects of chronic treatment. Indeed, we excluded at least 170 short-term studies during screening. Although antipsychotic agents differ in their metabolic risk profiles [8, 12, 14, 19], there was minimal evidence for antipsychotics other than risperidone in our review. Olanzapine, quetiapine, aripiprazole and ziprasidone are all used off-label in this population. As highlighted by Carnovale et al. [19], it is imperative to determine the nature of the dose–response relationship for each agent during prolonged use and if there are thresholds for adverse effects, as seen in adults [50, 51].

An important limitation of this review was the limited number of studies. Thus, we were unable to examine other factors contributing to or mitigating the effects of antipsychotics, such as age, prior treatment status, and other psychotropic medications. High heterogeneity amongst the included studies including different study designs and differing populations also prevented meta-analyses. Further, data were available for only two diagnostic disorders and thus we could not ascertain differences in outcomes by diagnosis. Most studies were reporting positive associations. It is possible there was publication bias and there are unpublished negative observational studies, especially since we did not cover grey literature.

## Conclusion

This systematic review highlights significant long-term risks for off-label antipsychotic treatment in children and adolescents, though existing evidence is limited. More controlled long-term studies are required to assess the implications for patients and the healthcare system. The use of real-world evidence from observational data in a target trial framework could provide immediate evidence for the cardiometabolic effects of long-term off-label use of a wide range of antipsychotics, which could possibly later be corroborated in clinical trials [52]. The benefits of symptom control in off-label indications needs to be weighed against the possibilities of adverse events on an individual basis. If treatment is required, it needs to be accompanied by psychosocial therapy and adequate monitoring for these metabolic effects. The possibility of antipsychotic discontinuation must be explored, along with early intervention for any adverse effects that arise from treatment.

## Supplementary Information

Below is the link to the electronic supplementary material.

Supplementary file1 (DOCX 47 KB)

Supplementary file2 (DOCX 1586 KB)

Supplementary file3 (XLSX 24 KB)

## Data Availability

No datasets were generated or analysed during the current study.
