# Supplementary material for: Cardiometabolic adverse effects of long-term antipsychotic treatment in children and adolescents with non-psychotic disorders: a systematic review of available evidence
Source: Eur Child Adolesc Psychiatry. 2025 Jun 5;34(11):3331–43. doi: 10.1007/s00787-025-02771-0 (PMC12647326; doi:10.1007/s00787-025-02771-0)
Supplement: Supplementary file 1 — Supplementary file1 (DOCX 47 KB) [file 787_2025_2771_MOESM1_ESM.docx]

# Supplementary Online Content

TITLE

Cardiometabolic adverse effects of long-term antipsychotic treatment in children and adolescents with non-psychotic disorders: A systematic review of available evidence

AUTHORS

Ramya Padmavathy Radha Krishnan^1^, Monika Dzidowska^1^, Danni Zheng^1^, Zoie Shui-Yee Wong^1,2^, Nicholas A Buckley^1^, Jacques Eugene Raubenheimer^1^

AFFILIATIONS

^1^Faculty of Medicine and Health, The University of Sydney, Sydney, New South Wales, Australia

^2^Graduate School of Public Health, St. Luke’s International University, Tokyo, Japan

Corresponding author

Ramya Padmavathy Radha Krishnan, Faculty of Medicine and Health, RC Mills Building Room 107, The University of Sydney, Sydney, New South Wales 2006, Australia

Email: [ramya.radhakrishnan@sydney.edu.au](mailto:ramya.radhakrishnan@sydney.edu.au)

**eTable 1** PICOT framework for eligibility criteria

| **Framework** | **Inclusion criteria** | **Exclusion criteria** |
| --- | --- | --- |
| Population | Patients of all age groups with non-psychotic illnesses:  obsessive-compulsive disorder, attention deficit hyperactivity disorder (ADHD), autism spectrum disorders, anxiety disorders (including neurotic disorder, panic disorder, phobias, separation anxiety, and social anxiety), sleep disorders (including insomnia, parasomnia and sleep wake disorder), dementia (including vascular and frontotemporal dementias, Huntington disease, Lewy body disease and cognition disorders) Alzheimer disease, agitation, personality disorders (including borderline, antisocial, compulsive, dependent, histrionic, paranoid and passive aggressive personality disorders, and hysterias), post-traumatic stress disorder (including adjustment disorder, battered child syndrome and stress disorder), intellectual disability, pervasive developmental disorder, learning disabilities, developmental disabilities, dyslexia, communication disorder, conduct disorders (including behaviour disorders, and aggression), dissociative disorders, substance abuse (alcohol, drugs, tobacco, and opioids), pathological gambling disorder, Tourette syndrome, chronic pain (including neuralgia, fibromyalgia and trigeminal neuralgia), headache, migraine, traumatic brain injury, Parkinson’s disease, self-injury, hiccup, delirium, mood disorders, chronic tic disorder, impulsive disorders (including gambling, trichotillomania, kleptomania, pyromania, and excoriation), non-psychotic depression (including major depressive disorder, dysthymic disorders, persistent depressive disorder, and disruptive mood dysregulation disorder), vomiting and nausea)  Patients with psychotic symptoms as an exacerbation of their psychiatric disorder were allowed | Patients with schizophrenia, schizoaffective disorder, bipolar disorder and psychotic depression  Pregnant women  Patients with eating disorders  Mixed population groups (containing both psychotic and non-psychotic patients) |
| Intervention | Treatment with any dose and method of administration of an antipsychotic, including combination treatments  (benperidol, chlorpromazine, chlorprothixene, droperidol, flupentixol, fluphenazine, fluspirilene, haloperidol, levomepromazine, loxapine, mesoridazine, molindone, periciazine, perphenazine, pimozide, prochlorperazine, promazine, penfluridol, perazine, spiperone, tiapride, trifluperidol, triflupromazine, sulpiride, thioproperazine, thioridazine, thiothixene, trifluoperazine, zuclopenthixol, amisulpride, aripiprazole, asenapine, blonanserin, brexpiprazole, cariprazine, clozapine, iloperidone, lurasidone, melperone, olanzapine, paliperidone, perospirone, quetiapine, remoxipride, risperidone, sertindole, ziprasidone, zotepine) | Lithium treatment as monotherapy |
| Comparator | Any, including placebo, other intervention and untreated patients | Studies without a comparator arm |
| Outcomes | Cardiometabolic adverse effects with measurable outcomes. Outcome measures given in parentheses.  Metabolic syndrome (>/= 1 metabolic syndrome criteria), hyperglycaemia (prediabetes, diabetes, insulin resistance, increased blood glucose, glucose intolerance), dyslipidemia (hypertriglyceridemia, hypercholesterolemia, changes in HDL, LDL or VLDL), weight gain (obesity, increase in adiposity, changes in BMI or waist/hip circumferences), hypertension (systolic and diastolic blood pressure), ischaemic heart disease, thrombosis (venous thromboembolism and cerebrovascular strokes) | All other adverse effects, including heart rate changes and arrhythmias |
| Time period | Long term treatment duration of 12 months or more (including mean duration for the cohort) | Short-term treatments lasting less than 12 months |
| Other | Language: English  Publication type: Peer-reviewed publications  Publication date: No limits  Study design: randomised clinical trials and observational studies in humans (cohort, cross-sectional and case-control). Cross-sectional studies with a comparator group and prior treatment duration >/= 12 months will be included. | Other language studies  Grey literature  None  All other publications including case reports, case series, letters, commentaries, opinions, editorials, unpublished data, trial protocols, studies without abstracts, before-after studies, methodology articles, reviews, and conference abstracts |

BMI: Body mass index, HDL: High-density lipoprotein cholesterol, LDL: Low-density lipoprotein cholesterol, VLDL: Very low-density lipoprotein cholesterol

**eTable 2** Final search strategy in MedLine

| **#No.** | **Terms** |
| --- | --- |
|  | **Antipsychotics (Group 1)** |
| 1 | exp Antipsychotic Agents/ or anti?psychotic*.mp. |
| 2 | Benperidol/ or (Anquil* or Frenact?l* or Glianimon* or Concilium or Benz?peridol* or Benzeridol or Benperidol* or Benquil).mp. |
| 3 | Chlorpromazine/ or (Aminazine or Chlorazine or Chlordelazine or Chlorpromazine* or Contomin or Fenactil or Largactil or Propaphenin or Thorazine).mp. |
| 4 | Chlorprothixene/ or (Taractan or Truxal or Chlorprothixen* or Chlorprotixen* or Chlothixen or Iaractan).mp. |
| 5 | Droperidol/ or (deh?drobenzperidol or droleptan or droperidol or inapsin* or Dridol or Properidol or Sintodril or Sintosian).mp. |
| 6 | Flupenthixol/ or (Flupenthixol or Flupentixol).mp. |
| 7 | Fluphenazine/ or (Flufenazin or Fluphenazin* or Lyogen or Prolixin).mp. |
| 8 | Fluspirilene/ or (Fluspiril?n* or Redeptin).mp. |
| 9 | Haloperidol/ or (haloperidol or Haldol or Serena?e or Aloperid* or Mixidol).mp. |
| 10 | Methotrimeprazine/ or (Levomeprazin or Levomepromazin* or Levopromazin* or Methotrimeprazin* or Ti?ercin* or Tizertsin).mp. |
| 11 | Loxapine/ or (Cloxazepine or Lox?pin* or Loxitane).mp. |
| 12 | Mesoridazine/ or (mesoridazin* or Serentil or Calodal or Lidanar or Lidanil).mp. |
| 13 | Molindone/ or (Moban or molindon*).mp. |
| 14 | Penfluridol/ or (Semap or Penfluridol* or Micefal).mp. |
| 15 | Perazine/ or Perazin*.mp. |
| 16 | (peric?azin* or properic?azin* or piperocyanomazine or Neuleptil or Nemactil or Neulactil or Aolept).mp. |
| 17 | Perphenazine/ or (Trilafon or Perphenazin* or Etaperazine or Perfenazin* or Ethaperazine or Fentazin or Chlorpiprazine or Perphenan or Thilatazin or Tranquisan or Decentan or Chlorperphenazine or Emesinal or Trifaron or Trilifan or Triphenot).mp. |
| 18 | Pimozide/ or (Orap or Opiran or Pimozid* or Primozid*).mp. |
| 19 | Prochlorperazine/ or (Prochlorperazin* or Prochlorpromazin* or Chlormeprazine or Chlorperazine or Capazine or Compazine or Prochlorpemazine or Meterazin* or Stemetil or Tementil or Kronocin or Emelent or Nipodal or Temetid or Novamin or Vertigon).mp. |
| 20 | Promazine/ or (Protactyl or Romtiazin or Sinophenin or Prazin* or Promazin* or Ampazin* or Berophen or Verophen or Esparin or Liranol or Sparine).mp. |
| 21 | Spiperone/ or (spiperon* or Spiropitan or Spiroperidol or Espiperona).mp. |
| 22 | (Ma?ept?l or thioproperazin* or Thioperazine or Sulfenazin or Tioproperazin* or Cephalmin or Megeptil or Vontil or Thiproperazin*).mp. |
| 23 | Thioridazine/ or (thioridazin* or Mell?ril or Meller?l or Meleril or Mallor?l or Sonapax or Orsanil or Mellarit or Thiozin*).mp. |
| 24 | Thiothixene/ or (Orbinamon or Navaron or Navan).mp. |
| 25 | Tiapride Hydrochloride/ or (Tiaprid* or Gramalil).mp. |
| 26 | Trifluoperazine/ or (Eskazin* or Jatroneural or Modalina or Stelazine or Stilizan or Terfluzine or Trifluoperaz* or Triftazin or Trifluperaz* or Trifluoroperaz* or Triflurin or Triperazin* or Trifluoromethylperazine or Flurazine or Triphthasine or Fluoperazine).mp. |
| 27 | Trifluperidol/ or (Trifluperidol* or Triperidol or Trised?l or Psychoperidol or Flumoperone or Psicoperidol).mp. |
| 28 | Triflupromazine/ or (triflupromazin* or Fluopromazin* or Trifluopromazin* or Vesprin or Adazine or Psyquil or Vetame).mp. |
| 29 | Clopenthixol/ or (Zuclopenthixol* or Clopixol or Zuclopentixol or Cisordinol or clopenthixol* or Zuclopentixol or Chlorpenthixol).mp. |
| 30 | Amisulpride/ or (amisulprid* or solian or deniban or Barn?til or Topral or Sultoprid*).mp. |
| 31 | Aripiprazole/ or (abilify* or Aripiprazol* or abilitat or aristada or aripiprex).mp. |
| 32 | (asenapine* or saphris or secuado).mp. |
| 33 | (blonanserin or lonasen).mp. |
| 34 | (brexpiprazole or r?xulti).mp. |
| 35 | (cariprazine* or vraylar or reagila).mp. |
| 36 | (Clozaril or FazaClo or Clozapin* or Zaponex or lepo?ex, iprox, asaleptin, klozapol).mp. |
| 37 | (iloperidon* or fanapt* or zomaril or fiapta).mp. |
| 38 | Lurasidone Hydrochloride/ or (lurasidon* or latuda).mp. |
| 39 | (melperon* or Bur?nil or eunerpan or Met?ylperon* or Harmosin or Libernal or Mel?Puren or Melneurin or flubuperon* or Bunil).mp. |
| 40 | Olanzapine/ or (olanzapin* or zyprexa* or zolafren or olansek or zalasta or zypadhera or olzapin or midax or oferta or lanzac).mp. |
| 41 | Paliperidone Palmitate/ or (paliperidon* or invega*).mp. |
| 42 | (perospiron* or lullan or perospirine).mp. |
| 43 | Quetiapine Fumarate/ or (quetiapin* or seroquel*).mp. |
| 44 | Remoxipride/ or (remoxiprid* or roxiam).mp. |
| 45 | Risperidone/ or (Risper?dal* or risperidon* or Rispolept or Risperin or Rispolin or Sequinan or Apexidone or Belivon or Psychodal or Spiron).mp. |
| 46 | (sertindol* or serdolect or serlect).mp. |
| 47 | Sulpiride/ or (sulp?ride or Aiglonyl or Arminol or Deponerton or Desisulpid or Digton or Dogmatil or Dolmatil or Eglonyl or Ekilid or Guastil or Lebopride or Meresa or neogama or Pontiride or Psicocen or Sulpitil or Sulpivert or Sulpor or Synedil or Tepavil).mp. |
| 48 | (ziprasidon* or Geodon or Zeldox or Zipradon).mp. |
| 49 | (zotepin* or nipolept or zoleptil or Lodopin or Engramon or Setous).mp. |
| 50 | 1 or 2 or 3 or 4 or 5 or 6 or 7 or 8 or 9 or 10 or 11 or 12 or 13 or 14 or 15 or 16 or 17 or 18 or 19 or 20 or 21 or 22 or 23 or 24 or 25 or 26 or 27 or 28 or 29 or 30 or 31 or 32 or 33 or 34 or 35 or 36 or 37 or 38 or 39 or 40 or 41 or 42 or 43 or 44 or 45 or 46 or 47 or 48 or 49 **(Group 1)** |
|  | **Off-label indications (Group 2)** |
| 51 | Attention Deficit Disorder with Hyperactivity/ or "attention deficit and disruptive behavior disorders"/ or (ADDH or ADHD).tw. or (attention adj deficit*).mp. or hyperactiv*.mp. or (oppositional defiant adj disorder).mp. |
| 52 | exp autism spectrum disorder/ or autistic disorder/ or (autism* or autistic*).mp. |
| 53 | exp Obsessive-Compulsive Disorder/ or (obsessive?compulsive* or body dysmorphi* or hoarding*).mp. or (obsessive adj compulsive).mp. or (compulsive adj behavio?r).mp. or OCD.tw. |
| 54 | "Sleep Initiation and Maintenance Disorders"/ or sleep wake disorders/ or dyssomnias/ or (insomnia or sleepless*).mp. or (sleep adj (disturb* or disorder*)).mp. |
| 55 | exp dementia/ or exp dementia, vascular/ or exp frontotemporal lobar degeneration/ or huntington disease/ or lewy body disease/ or cognition disorders/ or (dementia* or Lewy body* or Huntington* or cognition disorder).mp. |
| 56 | Alzheimer Disease/ or alzheimer*.mp. |
| 57 | Stress Disorders, Post-Traumatic/ or "trauma and stressor related disorders"/ or adjustment disorders/ or stress disorders, traumatic/ or battered child syndrome/ or PTSD.tw. or (post?traumatic or post traumatic).mp. or ((adjustment or stress) adj disorder).mp. or (battered child adj syndrome).mp. |
| 58 | personality disorders/ or antisocial personality disorder/ or borderline personality disorder/ or compulsive personality disorder/ or dependent personality disorder/ or histrionic personality disorder/ or hysteria/ or paranoid personality disorder/ or passive-aggressive personality disorder/ or (personality adj disorder*).mp. or hysteria.mp. |
| 59 | anxiety disorders/ or neurotic disorders/ or panic disorder/ or phobic disorders/ or Agoraphobia/ or (anxiety or anxious or neuros?s or neurotic or phobia* or agoraphobia or separation anxiety or social anxiety).mp. or ((panic or anxiety or neurotic or phobia) adj disorder*).mp. |
| 60 | Tourette Syndrome/ or tourette*.mp. |
| 61 | exp "feeding and eating disorders"/ or anorexia nervosa/ or binge-eating disorder/ or bulimia nervosa/ or diabulimia/ or food addiction/ or night eating syndrome/ or pica/ or ((feeding or eating) adj disorder*).mp. or (anorexi* or bulimi* or pica).mp. or (binge adj eating).mp. |
| 62 | Psychomotor Agitation/ or (agitat* or restless* or hyperactiv*).mp. |
| 63 | exp Intellectual Disability/ or exp child development disorders, pervasive/ or exp learning disabilities/ or exp communication disorders/ or developmental disabilities/ or dyslexia/ or ((mental* or intellectual* or learning) adj (deficienc* or retard* or disabilit* or disabled or challenged)).mp. or (challenging adj behavio?r).mp. or ((development* or communication) adj disorder).mp. |
| 64 | headache disorders/ or exp migraine disorders/ or (headache* or migraine*).mp. |
| 65 | exp Stroke/ or (stroke or cerebrovascular*).mp. |
| 66 | Conduct Disorder/ or Aggression/ or child behavior disorders/ or exp dissociative disorders/ or somatoform disorders/ or body dysmorphic disorders/ or body integrity identity disorder/ or hypochondriasis/ or ((conduct or behavio?r or dissociative or somatoform or body dysmorphic or body integrity or identity) adj disorder).mp. or (aggressive adj behavio?r).mp. or (aggression or aggressive or hypochondri*).mp. |
| 67 | exp "disruptive, impulse control, and conduct disorders"/ or firesetting behavior/ or gambling/ or trichotillomania/ or (trichotillomania* or gambling or fire?setting or kleptomania or arson or pyromania or hair-pulling* or hair pulling* or excoriation or skin-picking* or skin picking*).mp. or ((impulse?control or explosive or disrupt* or impuls*) adj2 (disorder or behavio?r)).mp. or (excoriat* adj disorder).mp. |
| 68 | exp Seizures/ or Epilepsy/ or (seizure* or convulsion* or epilepsy).mp. |
| 69 | Chronic Pain/ or Neuralgia/ or Trigeminal Neuralgia/ or (chronic adj pain).mp. or neuralgia*.mp. or (trigeminal adj neuralgia).mp. |
| 70 | brain injuries/ or exp brain hemorrhage, traumatic/ or exp brain injuries, diffuse/ or exp brain injuries, traumatic/ or exp brain injury, chronic/ or exp epilepsy, post-traumatic/ or (brain adj (injur* or laceration* or trauma or traumatic*)).mp. or TBI.tw. |
| 71 | substance-related disorders/ or alcoholic intoxication/ or alcoholism/ or amphetamine-related disorders/ or cocaine-related disorders/ or marijuana abuse/ or exp opioid-related disorders/ or phencyclidine abuse/ or substance abuse, intravenous/ or substance abuse, oral/ or ((substance or drug or alcohol or tobacco or amphetamine* or methamphetamine* or cocaine* or opioid* or opiate* or cannabis* or marijuana*) adj2 (abuse or addict* or intoxicat* or dependence)).mp. or alcoholism.mp. |
| 72 | Mood Disorders/ or Irritable Mood/ or (mood adj disorder*).mp. or irritabilit*.mp. or (irritable adj mood).mp. |
| 73 | parkinson disease/ or parkinson*.mp. |
| 74 | Hiccup/ or (hiccup* or hiccough*).mp. |
| 75 | Delirium/ or (delirium or disorient*).mp. |
| 76 | Self-Injurious Behavior/ or ((self-destructive or self-injur* or self-harm) adj behavio?r).mp. |
| 77 | Tics/ or Tic Disorders/ or tic.mp. or (tic adj disorder).mp. |
| 78 | Vomiting/ or Nausea/ or (vomiting or nausea or emesis).mp. |
| 79 | Depression/ or Depression.mp. or (major depressive adj disorder).mp. or Depressive Disorder, Major/ or (major adj depression).mp. or (disruptive mood dysregulation adj disorder).mp. or dysthymia.mp. or Dysthymic Disorder/ or (persistent depressive adj disorder).mp. |
| 80 | 51 or 52 or 53 or 54 or 55 or 56 or 57 or 58 or 59 or 60 or 61 or 62 or 63 or 64 or 65 or 66 or 67 or 68 or 69 or 70 or 71 or 72 or 73 or 74 or 75 or 76 or 77 or 78 or 79 **(Group 2)** |
|  | **Metabolic adverse effects (Group 3)** |
| 81 | (off?label or off indication or off-indication or un?approved or (off adj label)).mp. |
| 82 | "Drug-Related Side Effects and Adverse Reactions"/ or Pharmacovigilance/ or (adverse adj (effect* or event* or reaction or drug reaction or drug event)).mp. or (side effect* or tolerabilit* or safety or harm* or toxicit*).mp. |
| 83 | Metabolic Syndrome/ or (metabolic* or cardiometabolic* or dysmetabolic* or metabolic syndrome or metabolic abnormalit*).mp. |
| 84 | glucose metabolism disorders/ or diabetes mellitus/ or diabetes mellitus, type 2/ or diabetic ketoacidosis/ or latent autoimmune diabetes in adults/ or prediabetic state/ or hyperglycemia/ or insulin resistance/ or Blood Glucose/ or Glycated Hemoglobin A/ or Glycemic Control/ or (pre?diabet* or diabetes or insulin resistance or hyperglyc?emia or glucose or ketoacidosis or hba1c or glyc?emic control or glycated h?emoglobin).mp. or HOMA.tw. |
| 85 | dyslipidemias/ or hyperlipidemias/ or hyperlipoproteinemias/ or hypertriglyceridemia/ or Hypercholesterolemia/ or Cholesterol/ or Triglycerides/ or lipoproteins, hdl/ or exp cholesterol, hdl/ or lipoproteins, idl/ or exp lipoproteins, ldl/ or exp lipoproteins, vldl/ or (dyslipid?emia* or dyslipoprotein?emia* or hyperlipid?emia* or hypertriglycerid?emia or triglyceride or hypercholesterol?emia or cholesterol or blood lipid marker*).mp. or (HDL or LDL or VLDL).tw. |
| 86 | body weight/ or weight gain/ or exp overweight/ or exp obesity/ or anthropometry/ or body fat distribution/ or adiposity/ or body mass index/ or body weight/ or waist circumference/ or waist-height ratio/ or waist-hip ratio/ or (overweight or obesity or weight or waist circumference or hip circumference or body mass index or adiposity or obese or anthropometr*).mp. or BMI.tw. |
| 87 | hypertension/ or essential hypertension/ or prehypertension/ or Blood Pressure/ or (hypertension or blood pressure or systolic or diastolic).mp. or BP.tw. |
| 88 | Myocardial Ischemia/ or (isch?emic heart disease* or myocardial isch?emia*).mp. |
| 89 | thromboembolism/ or exp "intracranial embolism and thrombosis"/ or venous thromboembolism/ or thrombosis/ or coronary thrombosis/ or venous thrombosis/ or (thrombosis or thrombotic or pro?thrombotic).mp. |
| 90 | 81 or 82 or 83 or 84 or 85 or 86 or 87 or 88 or 89 **(Group 3)** |
| 91 | 50 and 80 and 90 **(Group 1 AND Group 2 AND Group 3)** |
| 92 | limit 90 to (english language and humans) |

**eTable 3** Title screening in EndNote

EndNote provides advanced grouping functionality such as custom groups, Smart Groups, Group Sets, and Combination Groups. These help to organise the references based on the search strategies and can be created from the Groups panel. The Clarivate Knowledge Hub* provides more information on the Grouping options in EndNote.

We used the grouping and Search functionalities to group the deduplicated references by various exclusion criteria parameters, as given in the table below. To ensure relevant literature is not eliminated, we removed references only after title screening and confirmation by a reviewer.

We applied these parameter filters in a sequential manner. Parameters and numbers for the initial search are given below (total unique references were 77,726). A similar pattern was observed in the regular updates. Note that some of the parameters overlap (for example, a reference could be grouped under article type as well as on-label) and thus numbers may add up to more than the total.

| **Parameter** | **Number of references** |
| --- | --- |
| Reviews | 20,041 |
| Other article types (Book, conference abstract, case report, chapter, comment, congress, dissertation, editorial, letter, newspaper, etc) | 27,303 |
| On-label (schizophrenia, schizoaffective, bipolar disorder, first-episode psychosis, psychotic depression) | 26,292 |
| Other psychotropics (antidepressants, lithium, ketamine, anti-epileptics, ADHD meds, etc) | 13,499 |
| Excluded conditions (cancer, surgery, palliative care, pregnancy) | 3,952 |
| Methodologically irrelevant (pharmacokinetics, pharmacodynamics, pharmacogenetics, drug interactions) | 2,733 |
| Preclinical studies (Animal models, invitro studies) | 1,069 |

* <https://support.clarivate.com/Endnote/s/?language=en_US>

<https://share.vidyard.com/watch/ndQyTmyYNLdQgbeN86StX3>

<https://support.clarivate.com/Endnote/s/article/FAQ-Groups?language=en_US>

**eTable 4:** List of indications in each arm of the studies with mixed psychiatric disorders

| **Study** | **Arm** | **Number of subjects** | **Indications*** |
| --- | --- | --- | --- |
| Calarge (USA, 2014) | Continuous risperidone | 74 | Disruptive behaviour disorder (92%), ADHD (88%), anxiety disorder (31%), depressive disorder (4%), autism spectrum disorder (16%) and tic disorder (23%). |
| Calarge (USA, 2014) | Antipsychotic switching | 9 | Disruptive behaviour disorder (78%), ADHD (78%), anxiety disorder (33%), depressive disorder (0%), autism spectrum disorder (22%) and tic disorder (33%). |
| Calarge (USA, 2014) | Discontinuation | 18 | Disruptive behaviour disorder (78%), ADHD (94%), anxiety disorder (28%), depressive disorder (11%), autism spectrum disorder (28%) and tic disorder (28%). |
| Calarge (USA, 2009) | Risperidone - overweight group | 34 | Disruptive behaviour disorder (65%), ADHD (82%), anxiety disorder (53%), depressive disorder (9%), tic disorder (24%), pervasive developmental disorder (24%), bipolar disorder (6%), psychotic disorder (0%). |
| Calarge (USA, 2009) | Risperidone - lean group | 65 | Disruptive behaviour disorder (65%), ADHD (91%), anxiety disorder (29%), depressive disorder (25%), tic disorder (18%), pervasive developmental disorder (12%), bipolar disorder (2%), psychotic disorder (3%). |
| Wei et al (USA, 2017) | Short-term AP use | 1794 | *Indications in the entire cohort (all 3 arms):*  Autism (49.1%), ADHD (44.7%), mood disorder (10.3%), bipolar disorder (7.5%), oppositional defiant disorder (7.5%), developmental delay, intermittent explosive disorder or behaviour disorder (7.5%), Disruptive behaviour (6.3%), tics (2.1%) |
| Wei et al (USA, 2017) | Long-term AP use |  |  |
| Wei et al (USA, 2017) | Continuous AP use >2 years |  |  |
| Roke et al (Netherlands, 2012) | Multiple APs | 56 | Autism spectrum disorder (93%) or disruptive behaviour disorder (7%) |
| Roke et al (Netherlands, 2012) | AP-untreated patients | 47 | Autism spectrum disorder (85%) or disruptive behaviour disorder (15%) |
| Lee et al (Korea, 2018) | T2DM cases | 20263 | Anxiety disorder (21.29%), depression (13.75%), ADHD (10.64%), somatoform disorder (8.7%), reaction to severe stress and adjustment disorder (7.49%), other neurotic disorders (7.3%), Mental retardation (4.79%), other behaviour and emotional disorders with onset usually occurring in childhood and adolescents (3.22%), emotional disorders with onset specific to childhood (2.56%), nonorganic disorder (2.49%), tic disorder (1.87%), bipolar disorder (0.52%), schizophrenia spectrum (0.43%), other psychiatric disorders (14.95%) |
| Lee et al (Korea, 2018) | Non-T2DM controls | 80043 | Anxiety disorder (21.54%), depression (13.9%), ADHD (10.77%), somatoform disorder (8.81%), reaction to severe stress and adjustment disorder (7.58%), other neurotic disorders (7.39%), mental retardation (4.58%), other behavior and emotional disorders with onset usually occurring in childhood and adolescents (3.26%), emotional disorders with onset specific to childhood (2.59%), nonorganic disorder (2.52%), tic disorder (1.87%), bipolar disorder (0.49%), schizophrenia spectrum (0.28%), other psychiatric disorders (14.42%) |

* indications may not add up to 100% due to psychiatric comorbidities. Comorbidities also account for the presence of schizophrenia and bipolar disorder among the list of indications.

**eTable 5:** List of comedications administered in each arm of the studies

| **Study** | **Arm** | **Concomitant treatments (% of subjects administered)** |
| --- | --- | --- |
| Calarge (USA, 2014) | Continuous risperidone | Concomitant medications at the end of follow-up:  psychostimulants (80%), alpha-2 agonists (34%), antidepressant (58%) and mood stabilizers (8%). |
| Calarge (USA, 2014) | Antipsychotic switching | Concomitant medications at the end of follow-up:  psychostimulants (56%), alpha-2 agonists (67%), antidepressant (89%) and mood stabilizers (0%). |
| Calarge (USA, 2014) | Discontinuation | Concomitant medications at the end of follow-up:  psychostimulants (61%), alpha-2 agonists (28%), antidepressant (56%) and mood stabilizers (11%). |
| Degrauw et al (USA, 2009) | Multiple APs | Stimulant without SSRI (37.5%), SSRI without stimulant (31.25%), stimulant and SSRI (12.5%), no stimulant no SSRI (18.75%). Use of alpha-adrenergic agonists was common. |
| Degrauw et al (USA, 2009) | AP-untreated patients | Stimulant without SSRI (20.68%), SSRI without stimulant (24.13%), stimulant and SSRI (6.89%), no stimulant no SSRI (48.27%). Use of alpha-adrenergic agonists was common. |
| Yoon et al (USA, 2016) | Risperidone | Stimulant (6.8%), Metformin (3.4%), Topiramate (0), Antiepileptic drug other than topiramate (3.4%), SSRI/SNRI (11.9%), alpha-2-agonist (22%), benzodiazepine (0), Other (28.8%), None (40.7%) |
| Yoon et al (USA, 2016) | Aripiprazole | Stimulant (12.1%), Metformin (1.5%), Topiramate (1.5%), Antiepileptic drug other than topiramate (1.5%), SSRI/SNRI (4.5%), alpha-2-agonist (16.7%), benzodiazepine (0), Other (22.7%), None (43.9%) |
| Yoon et al (USA, 2016) | Olanzapine | Stimulant (0), Metformin (7.1%), Topiramate (0), Antiepileptic drug other than topiramate (28.6%), SSRI/SNRI (35.7%), alpha-2-agonist (7.1%), benzodiazepine (7.1%), Other (35.7%), None (28.6%) |
| Yoon et al (USA, 2016) | Quetiapine | Stimulant (10%), Metformin (0), Topiramate (0), Antiepileptic drug other than topiramate (13.3%), SSRI/SNRI (30%), alpha-2-agonist (20%), benzodiazepine (10%), Other (46.7%), None (20%) |
| Yoon et al (USA, 2016) | Ziprasidone | Stimulant (9.1%), Metformin (0), Topiramate (0), Antiepileptic drug other than topiramate (6.1%), SSRI/SNRI (30.3%), alpha-2-agonist (18.2%), benzodiazepine (9.1%), Other (27.3%), None (27.3%) |
| Ondo et al (USA, 2008) | Multiple APs | Entire period - SNRI (26.8%), stimulant (19.5%) Patrial period - SNRI (31.7%), stimulant (17.0%) |
| Ondo et al (USA, 2008) | Tetrabenazine | Entire period - SNRI (55.6%), stimulant (22.2%) Partial period - SNRI (22.2%), stimulant (5.5%) |
| Wink et al (USA, 2014) | Risperidone | SSRI (28%), antiepileptic (7%), stimulant (21%), metformin (6%), alpha-agonist (37%), benzodiazepine (0%), other (36%), none (26%) |
| Wink et al (USA, 2014) | Aripiprazole | SSRI (30%), antiepileptic (6%), stimulant (14%), metformin (3%), alpha- agonist (31%), benzodiazepine (3%), other (34%), none (29%) |
| Rizzo et al (Italy, 2012) | Aripiprazole | Fluoxetine (40%), biperiden cloridrate (28%) |
| Rizzo et al (Italy, 2012) | Pimozide | Fluoxetine (28%), biperiden cloridrate (48%) |
| Calarge (USA, 2009) | Risperidone - overweight group | Psychostimulants (50%), alpha-2 agonists (47%), SSRI (53%) |
| Calarge (USA, 2009) | Risperidone - lean group | Psychostimulants (77%), alpha-2 agonists (25%), SSRI (52%) |
| Wei et al (USA, 2017) | Short-term AP use | Psychotropics at baseline in the entire cohort:  ADHD medications (67.5%), anticonvulsants, antidepressants (8.8%), anxiolytic, hypnotics and sedatives (9.7%) |
| Wei et al (USA, 2017) | Long-term AP use | Psychotropics at baseline in the entire cohort:  ADHD medications (67.5%), anticonvulsants, antidepressants (8.8%), anxiolytic, hypnotics and sedatives (9.7%) |
| Wei et al (USA, 2017) | Continuous AP use >2 years | Psychotropics at baseline in the entire cohort:  ADHD medications (67.5%), anticonvulsants, antidepressants (8.8%), anxiolytic, hypnotics and sedatives (9.7%) |
| Roke et al (Netherlands, 2012) | Multiple APs | Melatonin (9%), SSRI (4%), psychostimulants (18%), atomoxetine (4%) |
| Roke et al (Netherlands, 2012) | AP-untreated patients | Melatonin (9%), SSRI (0%), psychostimulants (28%), atomoxetine (0%) |
| Vanwong et al (Thailand, 2020) | Risperidone | Concomitant medications were given to 50% of the patients and included aripiprazole (1.5%), diphenhydramine (1.5%), methylphenidate (77.6%), fluoxetine (9%), folic acid (1.5%), atomoxetine (3%), sertraline (4.5%) and topiramate (1.5%). |
| Vanwong et al (Thailand, 2020) | General population | Not given |
| Croteau et al (Canada, 2019) | Obese cases | Antidepressants (7.4%), anticonvulsants (3%), ADHD drugs (5.9%) |
| Croteau et al (Canada, 2019) | Non-obese controls | Antidepressants (3.6%), anticonvulsants (3.4%), ADHD drugs (3.8%) |
| Lee et al (Korea, 2018) | T2DM cases | Antidepressants (6.87%), Benzodiazepine (8.03%), typical antipsychotics (2.38%) |
| Lee et al (Korea, 2018) | Non-T2DM controls | Antidepressants (6.36%), Benzodiazepine (7.09%), typical antipsychotics (1.58%) |

SSRI: selective serotonin reuptake inhibitor, SNRI: serotonin/norepinephrine reuptake inhibitor,
ADHD: attention deficit hyperactivity disorder
