# Supplementary material for: Cardiometabolic adverse effects of long-term antipsychotic treatment in children and adolescents with non-psychotic disorders: a systematic review of available evidence
Source: Eur Child Adolesc Psychiatry. 2025 Jun 5;34(11):3331–43. doi: 10.1007/s00787-025-02771-0 (PMC12647326; doi:10.1007/s00787-025-02771-0)
Supplement: Supplementary file 2 — Supplementary file2 (DOCX 1586 KB) [file 787_2025_2771_MOESM2_ESM.docx]

# Supplementary Online Content

TITLE

Cardiometabolic adverse effects of long-term antipsychotic treatment in children and adolescents with non-psychotic disorders: A systematic review of available evidence

AUTHORS

Ramya Padmavathy Radha Krishnan^1^, Monika Dzidowska^1^, Danni Zheng^1^, Zoie Shui-Yee Wong^1,2^, Nicholas A Buckley^1^, Jacques Eugene Raubenheimer^1^

AFFILIATIONS

^1^Faculty of Medicine and Health, The University of Sydney, Sydney, New South Wales, Australia

^2^Graduate School of Public Health, St. Luke’s International University, Tokyo, Japan

Corresponding author

Ramya Padmavathy Radha Krishnan, Faculty of Medicine and Health, RC Mills Building Room 107, The University of Sydney, Sydney, New South Wales 2006, Australia

Email: [ramya.radhakrishnan@sydney.edu.au](mailto:ramya.radhakrishnan@sydney.edu.au)

**eFig. 1** Study selection

150,244 studies identified

Embase=73,585

Scopus=24,165

Medline=21,607

PsycInfo=10,780

CINAHL=10,074

Web of Science=8,097

Reviews and similar articles=1,936

92,381 titles preliminary screening

Identification

Screening

744 reports sought for

full-text screening

727 full-text reports

assessed for eligibility

30 studies included

16 reports on children and adolescents

Included

57,863 duplicates removed

87,597 studies excluded

17 full-text reports not available

697 full-texts excluded:

Shorter duration=174

Mixed population=155

Wrong study design=115

Psychotic/no indication=106

Wrong outcomes=73

No comparator=57

Other interventions=12

Other language=5

14 reports on adults and elderly

4,784 abstracts screened

4,040 abstracts excluded

Preliminary screening was performed using SmartGroup filters and Search functionality in EndNote. Abstract and full-text screening were carried out in Covidence.

**eFig. 2** Data availability for antipsychotic agents and outcomes


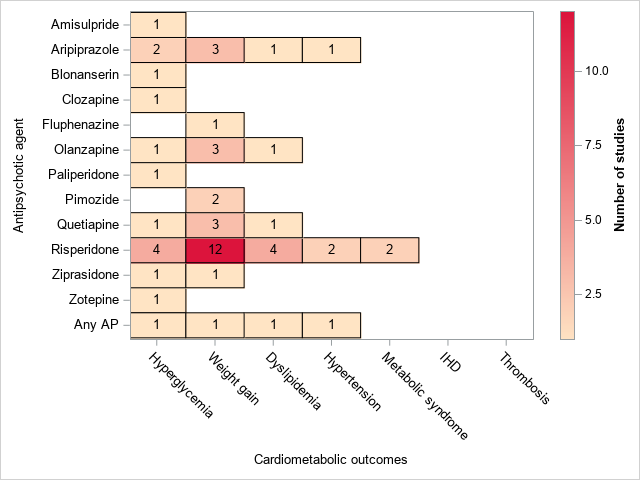


The number of studies for each outcome is represented in a matrix against the individual antipsychotics studied. Studies examining multiple antipsychotics were counted individually for each antipsychotic where possible.

AP: Antipsychotic, IHD: Ischaemic heart disease

**eFig. 3** Study quality assessment by study type for the included studies


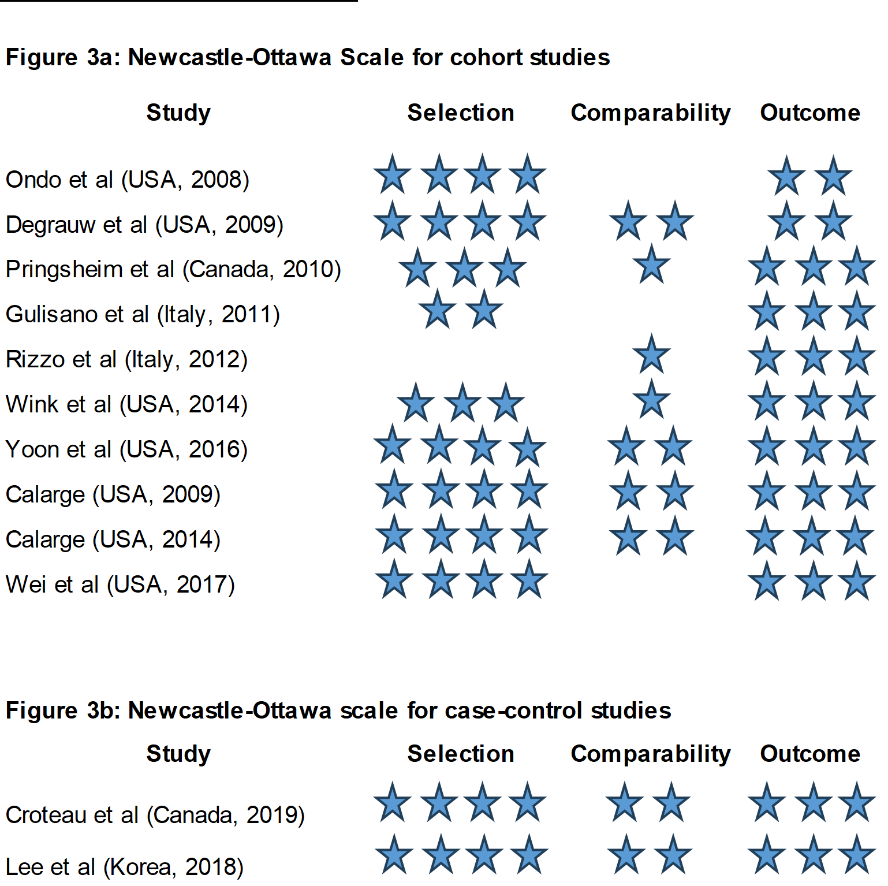

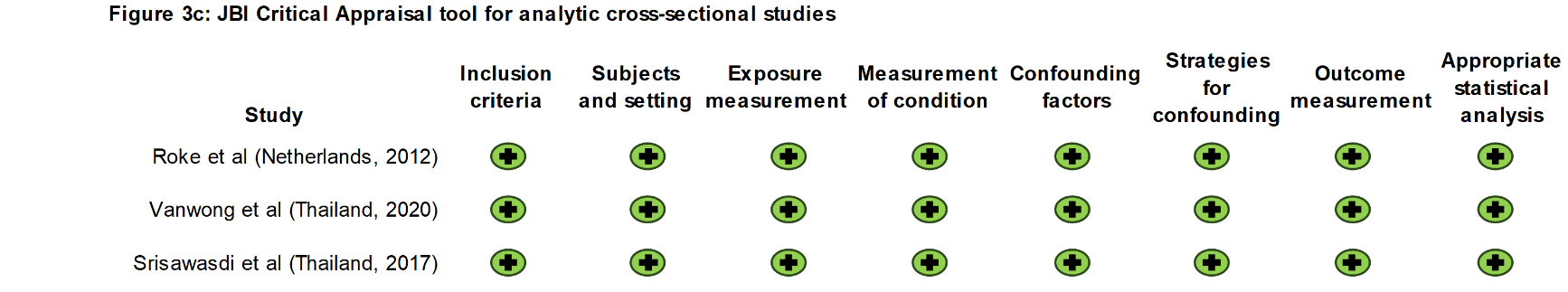


1a) The Newcastle-Ottawa scale for cohort studies, with a maximum of four stars for Selection, two for Comparability and three stars for Outcome

1b) The Newcastle-Ottawa scale for case-control studies, with a maximum of four stars for Selection, two for Comparability and three stars for Outcome

1c) The Joanna-Briggs Institute (JBI) tool for analytic cross-sectional studies has eight questions with answers of

Yes


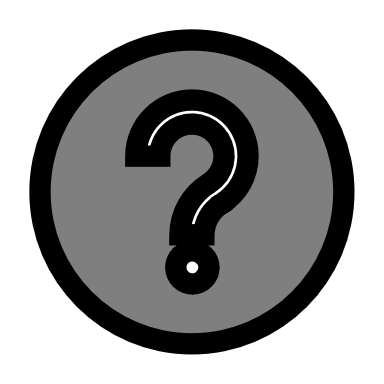
No

Unclear

Not applicable
